# FDG PET/CT reveals bone marrow oligometastasis in laryngeal squamous carcinoma: a case report with favorable outcome

**DOI:** 10.1259/bjrcr.20230065

**Published:** 2023-10-26

**Authors:** Akram Al-Ibraheem, Dhuha Ali Al-Adhami, Ahmed Saad Abdlkadir, Issa Mohamad, Hamza Ghatasheh, Monther Qandeel

**Affiliations:** 1 Department of Nuclear Medicine and PET/CT, King Hussein Cancer Center, Al-Jubeiha, Amman, Jordan; 2 Department of Radiology and Nuclear Medicine, Division of Nuclear Medicine, University of Jordan, Amman, Jordan; 3 Department of Radiation Oncology, King Hussein Cancer Center, Jordan Al-Jubeiha, Amman, Jordan; 4 Department of Diagnostic Radiology, King Hussein Cancer Center, Jordan Al-Jubeiha, Amman, Jordan

## Abstract

Laryngeal carcinoma is the most common head and neck cancer. The vast majority of laryngeal carcinomas are of squamous-cell histologic type. Metastasis of laryngeal cancer typically occurs within the cervical lymph nodes and seldom in other regions. Although a small percentage of patients experience distant metastases, bone marrow metastasis from laryngeal cancer is among the least common metastatic sites. Previous literature has suggested that bone marrow carcinomatosis is aggressive and has a poor outcome, particularly in patients with supraglottic tumors. Ante-mortem diagnosis of this metastatic pattern has been limited. To our knowledge, this case report highlights the first documented occurrence wherein the utilization of 18-fluorine fludeoxyglucose positron emission tomography/CT imaging played a pivotal role in the early detection of bone marrow metastasis in a patient diagnosed with transglottic laryngeal cancer. A solitary metastatic distant bone marrow lesion was identified early during follow-up. As a consequence, the patient exhibited a remarkable and unforeseen favorable clinical outcome.

## Clinical presentation

A 61-year-old male patient with a prolonged history of smoking presented with a 1-month history of voice hoarseness, and throat pain. The rapid progress of worsening pain and hoarseness in voice was worrisome and warranted further diagnostic work-up.

## Investigations, outcome follow-up

**Figure 1.**
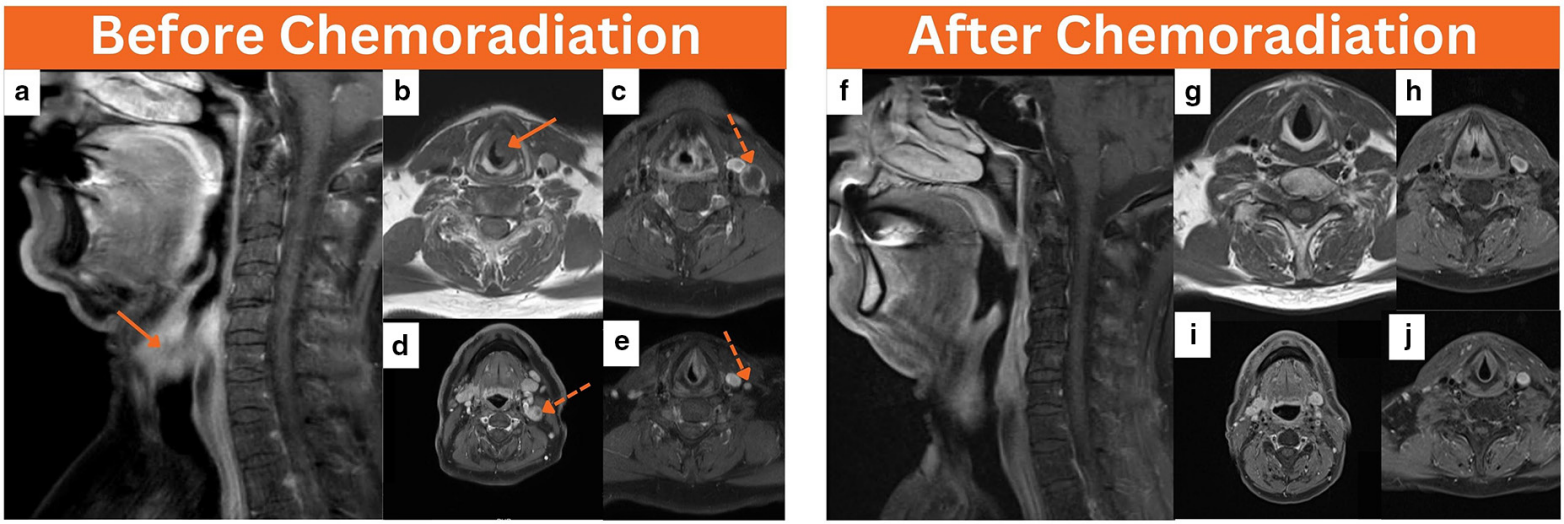
Baseline neck MRI was performed before chemoradiation, coronal T1 neck MRI (**a**) and axial T1 images (**b**) revealed a left transglottic laryngeal mass infiltrating the left paraglottic space and measuring about 2.5 cm in maximum dimension (arrows). In addition, axial T1 images of the neck (c–e) showed few concurrent ipsilateral enlarged level II–IV cervical lymph nodes, the majority of which demonstrate central necrosis (dotted arrows). At the end of therapy, coronal T1 neck MRI (**f**) and axial T1 neck images (g–j) concluded resolution of the previous locoregional disease.

**Figure 2.**
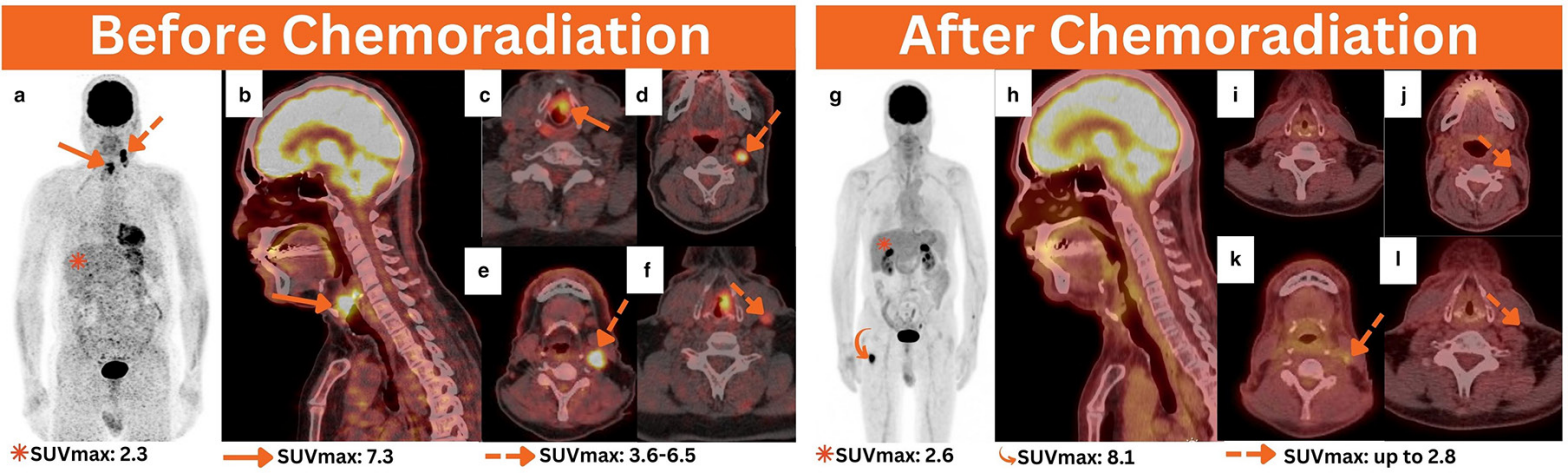
Baseline ^18^F-FDG PET/CT performed before chemoradiation for staging purposes. MIP (**a**), sagittal (**b**), and axial ^18^F-FDG PET/CT images (**b, c**) revealed hypermetabolic left glottic malignancy (arrows). Additionally, axial PET/CT images of the neck (e–f) showed a few ipsilateral hypermetabolic cervical lymphadenopathies involving II–IV levels (dotted arrows). In follow-up, ^18^F-FDG PET/CT performed after chemoradiation for response assessment, MIP (**g**), sagittal (**h**), and axial PET/CT images (i–l) revealed evidence of complete resolution in the previous local disease. This was, however, complicated by a distant hypermetabolic intramedullary focus occupying the right proximal femur, as evidenced by the MIP image, necessitating further evaluation (curved arrow). ^18^F-FDG PET/CT, 18-fluorine fludeoxyglucose positron emission tomography/CT; MIP, maximum intensity projection.

**Figure 3.**
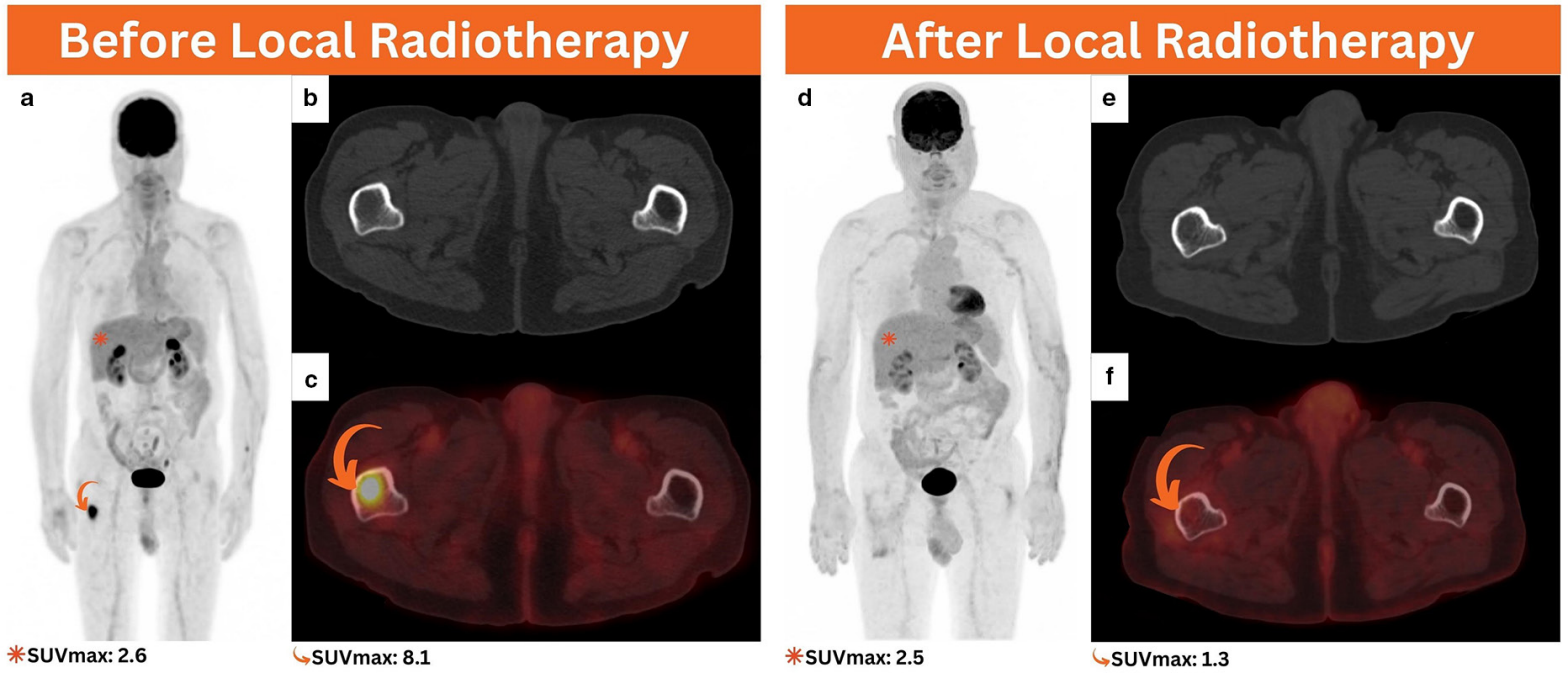
Follow-up ^18^F-FDG PET/CT performed after the initial chemoradiation for response assessment, MIP (**a**), axial CT (**b**), and axial PET/CT (**c**) revealed evidence of a small intramedullary hypermetabolic focus involving the subtrochanteric region of the right femur without significant osseous abnormality on the axial CT images (curved arrows). 3 months following local radiation treatment, ^18^F-FDG PET/CT was performed for response assessment, MIP (**d**), axial CT (**e**), and axial PET/CT (**f**) denoted evidence of a complete metabolic response in the irradiated region (curved arrow). ^18^F-FDG PET/CT, 18-fluorine fludeoxyglucose positron emission tomography/CT; MIP, maximum intensity projection.

**Figure 4.**
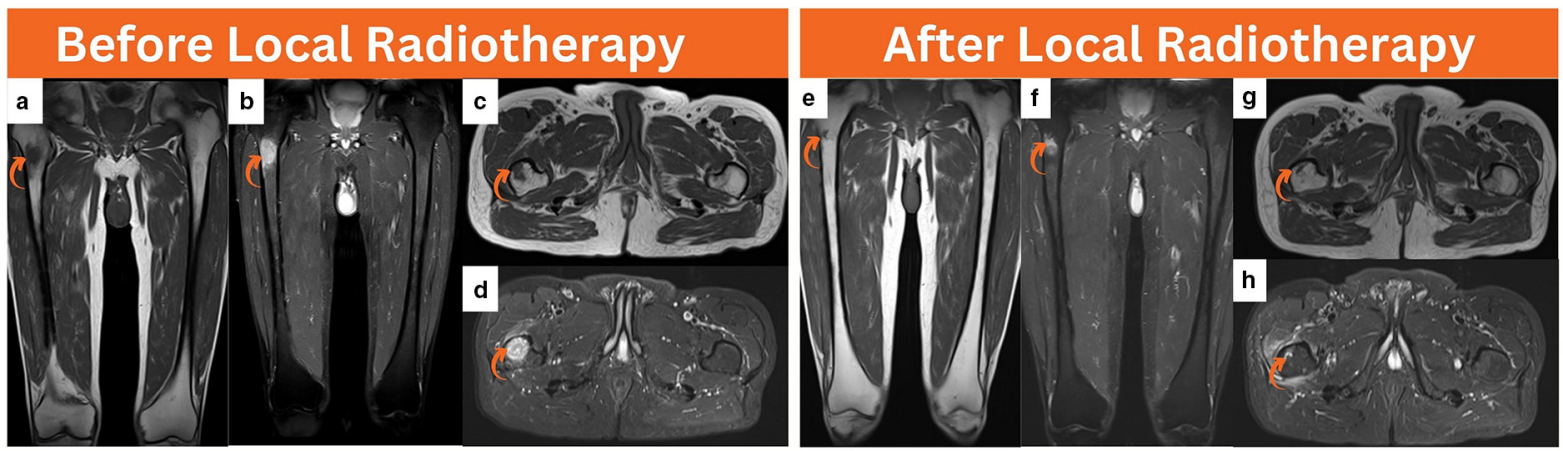
Lower extremity MRI performed after interval development of a distant unifocal right subtrochanteric focus detected by ^18^F-FDG PET/CT for imaging correlation, coronal T1 (**a**), coronal T2 (**b**), axial T1 (**c**), and axial T2 (**d**) images revealed a small intramedullary subtrochanteric lesion demonstrating hypointensity on T1 images and moderate hyperintensity on T2 images measuring about 1.4 cm in maximum dimension (curved arrow). Following local radiotherapy, follow-up lower extremity MRI was performed, and coronal T1 (**e**), coronal T2 (**f**), axial T1 (**g**), and axial T2 (**h**) images denoted evidence of near complete healing in the previous right subtrochanteric lesion (curved arrow). ^18^F-FDG PET/CT, 18-fluorine fludeoxyglucose positron emission tomography/CT.

Initial laboratory tests, including liver function test (LFT), renal function test (RFT), a complete blood count (CBC), and a chest radiographs, were normal. As a result, MRI was conducted, which identified a left laryngeal transglottic mass that had not invaded the thyroid or cricoid cartilage and demonstrated no extralaryngeal extension (Figure 1a and b, arrows). Additionally, there were a few enlarged ipsilateral lymph nodes demonstrating central necrosis (Figure 1c–e, dotted arrows). 18-fluorine fludeoxyglucose positron emission tomography/CT (^18^F-FDG PET/CT) scan were ordered for staging (Figure 2a–f). The study showed a hypermetabolic malignant left transglottic lesion (Figure 2a–c, arrows), associated with a few hypermetabolic ipsilateral metastatic levels II and III cervical lymph nodes (Figure 2d–f, dotted arrows). Histopathological examination confirmed the diagnosis of poorly differentiated squamous cell carcinoma (SCC) of the left glottis. Overall, staging results were indicative of T2, N2_b_, and M0 disease staged as IVA disease according to AJCC eighth edition. The patient’s scenario was discussed in a multidisciplinary clinic (MDC). Based on the MDC’s decision, the patient commenced concomitant chemoradiotherapy (CCRT). The patient received a total of 6 cycles of chemotherapy and 35 radiotherapy fractions. Following the third cycle of chemotherapy, the patient manifested sudden swelling of his right leg which rendered him unable to ambulate using the affected limb, prompting his presentation to the emergency department in a wheelchair. Upon clinical examination, right leg redness, hotness and tenderness was observed raising the suspicion of deep venous thrombosis (DVT). Subsequent investigation with doppler ultrasound revealed extensive venous thrombosis affecting the superficial femoral vein, popliteal and posterior tibial veins. The patient was consequently placed on anticoagulant therapy to manage the DVT, while orthopedic care was provided through the use of crutches for assistance with walking. At the end of therapy, neck MRI was performed 3 months following CCRT and showed dramatic improvement of the known left laryngeal tumor and left cervical lymphadenopathy (Figure 1f–j). Concurrently, an ^18^F-FDG PET/CT scan showed complete resolution of the previous hypermetabolic locoregional disease (Figure 2g–l). This was, however, complicated by a distant hypermetabolic intramedullary focus in the right proximal femur (Figure 3a–c, curved arrows). Notably, during the ^18^F-FDG PET/CT imaging, the patient did not disclose any incidence of new-onset pain at the right thigh location, nor did he exhibit any discernible escalation in pain or tenderness at the DVT site. During this time and beyond, alkaline phosphatase (ALP) levels remained within normal ranges. The evidence from the whole-body ^18^F-FDG PET/CT report was alarming for potential bone marrow involvement and necessitated further evaluation. Therefore, MRI of both thighs was performed exactly 2 days after whole-body ^18^F-FDG PET/CT to further explore and identify this lesion. The MRI study confirmed the presence of an intramedullary lesion in the right femoral subtrochanteric region with perilesional edema (Figure 4a–d, curved arrows). The patient underwent five fractions of localized radiotherapy to the right femur, and follow-up scans showed rapid regression of the lesion and only minimal post-radiotherapy changes (Figure 3d–f, Figure 4e–h). During the course of illness, CBC, LFT and RFT demonstrated no significant abnormalities. To date, the patient has remained in remission and maintained close follow-up for the past 22 months.

## Discussion

The global occurrence of HNC is on the rise.^1^ In HNC, the overall incidence of distant metastases is reported to be around 8.5% in cancers of the larynx, with the lung being the most commonly affected site, followed by the bones and liver.^2^ Notably, bone marrow metastases are not frequently recognized. In fact, only a few cases of laryngeal cancer with bone marrow metastases have been reported.^3–5^ Factors that was observed to increase the likelihood of distant metastases include locally advanced primary tumors, nodal metastatic disease, poor differentiation, and malignant supraglottic localization.^4,5^ Hong and colleagues examined the recurrence patterns in 103 patients with locally advanced HNC who had previously achieved complete remission.^4^ The study concluded that patients with extensive tumors are more likely to develop distant metastases, and that CCRT does not offer protection against future metastasis.^4^ As a result, close monitoring is necessary for advanced HNC patients to detect any potential metastatic relapse.

The underdiagnosis of metastatic disease in laryngeal cancer is a well-known issue.^6^ In fact, distant metastases can be found in patients who were previously thought to be cured and showed no clinical signs of cancer.^6^ Two examples have been reported as such previously in a case series by Narula et al.^6^ The occurrence of bone marrow metastases is attributed to hematogenous cancer spread at early stages of tumor progression.^7^ Initially, it can be challenging to identify this through clinical and conventional imaging methods.^7^ Days or months later, the infiltration of bone marrow can lead to the progression of either macrometastasis within the bone marrow or the formation of osseous metastases.^7^

In the previous literature, the majority of patients experiencing bone marrow metastases display symptoms associated with bone marrow infiltration, including hemoptysis and ecchymosis.^8^ Laboratory tests generally reveal anemia due to impaired hematopoiesis. As the condition progresses, severe thrombocytopenia, pancytopenia, or disseminated intravascular coagulopathy may occur,^9^ which could be confused with chemotherapy complications. However, our patient did not exhibit any of these symptoms, possibly due to early detection and unifocality. In cases where bone marrow metastasis is limited and confined to a single location, the primary clinical presentation commonly involve localized symptoms. These symptoms include local bone pain, which is non-specific and difficult to pinpoint.^8^ Furthermore, nerve entrapment in specific areas, such as vertebral metastasis, can result in the emergence of sensory abnormalities including numbness and parasthesia.^10^ Patients harboring a lesion within peripheral skeleton may also encounter restricted mobility or, in severe cases, a loss of function on the affected side due to noticeable discomfort.^11^ The first documented instance of a diagnosis of bone marrow metastases from laryngeal cancer prior to death was reported in 1989.^5^ Following the third cycle of cytotoxic chemotherapy, the patient experienced pancytopenia, which was mistakenly attributed to excessive alcohol consumption and chemotherapy, rather than being considered in the context of bone marrow metastases.^5^ Diagnostic procedures carried out 2 months later confirmed fulminant bone marrow and liver metastases, and the patient died soon afterwards.^5^ By contrast, in our case report, the patient’s CBC levels remained stable throughout his illness because of an early diagnosis of focal bone marrow metastases by whole body ^18^F-FDG PET/CT. In addition, our case did not show an increase in ALP levels during the illness. This can be attributed to the inadequate accuracy of this marker.^12^ Nevertheless, recent research has indicated the advantages of having a negative ALP in metastatic HNC. It has been discovered that a negative ALP indicates a favorable outcome for HNC.^13^ Therefore, having negative ALP marker levels does not exclude the potential of having bone marrow metastases but can indicate a favorable outcome in the long run.

When a patient presents with a solitary lesion in the femoral bone marrow, radiologists are required to consider a diverse range of potential diagnoses. This includes primary bone tumors, such as osteosarcoma, which may exhibit comparable imaging characteristics.^14^ Moreover, it is imperative to exclude the existence of recently formed primary neoplasms derived from the hematologic system, such as lymphoma.^14^ Furthermore, it is important to consider the potential occurrence of a secondary primary HNC or other solid tumors infiltrating the bone marrow.^14^ Lastly, a meticulous evaluation is necessary in order to differentiate between inflammatory conditions such as osteomyelitis and malignant tumors,^14^ as they may exhibit similar imaging features.^18^F-FDG PET/CT is an essential tool for diagnosing and evaluating response in metastatic laryngeal squamous carcinoma.^15^ It outperforms MRI in detecting and assessing nodal and metastatic disease.^16^ The overall sensitivity and specificity ranks above 90% in detecting metastases.^17^ Therefore, utilizing ^18^F-FDG PET/CT for a whole-body examination can provide a comprehensive assessment and identify any potential distant bone or bone marrow metastases, regardless of whether they are widespread or limited in number (oligometastatic).

To date, there is limited evidence regarding the management of unifocal oligometastatic disease.^18^ The term oligometastatic disease was first introduced in 1995 to describe patients who have less than five metastatic foci.^19^ Bates et al conducted a study which found that oligometastatic HNC patients who received stereotactic body radiation had positive outcomes with good local control.^20^ Thus, timely detection and effective management are crucial factors in improving disease-specific and survival outcomes. The strategy mentioned can be effectively executed by using a whole-body protocol for FDG PET/CT imaging.^21^ A crucial factor to keep in mind when using FDG PET/CT after treatment is to decrease false-positive results. To achieve this, it is strongly recommended to schedule FDG PET/CT scans between 3 and 6 months after CCRT, rather than immediately after the completion of treatment.^22^

## Conclusion

In all previous reported cases, bone marrow metastases were either misidentified, undiagnosed, or diagnosed too late resulting in significant complications and unfavorable outcomes. It is therefore crucial to increase awareness of similar cases, advocate for the implementation of whole-body molecular imaging, and closely monitor patients with high clinicopathological characteristics to achieve early diagnosis, improved management, and favorable outcomes.

## Learning points

- The incidence of HNCs is increasing.
- ^18^F-FDG PET/CT imaging is highly valuable in the detection, evaluation and response assessment of nodal, metastatic, and synchronous pathologies.
- Whole-body ^18^F-FDG PET/CT imaging must be offered routinely to exclude the possibility of distant metastatic spread and ensure accurate response assessment.
- Patients with oligometastatic disease can benefit from local radiotherapy sessions, particularly if effective control of the local disease is achieved.
